# Multilayer Film Comprising Polybutylene Adipate Terephthalate and Cellulose Nanocrystals with High Barrier and Compostable Properties

**DOI:** 10.3390/polym16152095

**Published:** 2024-07-23

**Authors:** Beatriz Melendez-Rodriguez, Cristina Prieto, Maria Pardo-Figuerez, Inmaculada Angulo, Ana I. Bourbon, Isabel R. Amado, Miguel A. Cerqueira, Lorenzo M. Pastrana, Loic Hugues Gilles Hilliou, António A. Vicente, Luis Cabedo, Jose M. Lagaron

**Affiliations:** 1 Novel Materials and Nanotechnology Group, Institute of Agrochemistry and Food Technology (IATA), Spanish Council for Scientific Research (CSIC), Calle Catedrático Agustín Escardino Benllonch 7, 46980 Valencia, Spain; beatriz.melendez@iata.csic.es (B.M.-R.); cprieto@iata.csic.es (C.P.); mpardo@iata.csic.es (M.P.-F.); 2 Bioinicia R&D, Bioinicia S.L., Calle Algepser 65, Nave 3, 46980 Paterna, Spain; 3 Gaiker Technological Centre, Department of Plastics and Composites, Parque Tecnológico Edificio 202, 48170 Zamudio, Spain; angulo@gaiker.es; 4 International Iberian Nanotechnology Laboratory (INL), Av. Mestre José Veiga s/n, 4715-330 Braga, Portugal; ana.bourbon@inl.int (A.I.B.); isabel.rodriguez@inl.int (I.R.A.); miguel.cerqueira@inl.int (M.A.C.); lorenzo.pastrana@inl.int (L.M.P.); 5 IPC/I3N, Department Polymer Engineering, Campus de Azurém, 4800-058 Guimarães, Portugal; loic@dep.uminho.pt; 6 Centre of Biological Engineering, University of Minho, Campus Gualtar, 4710-057 Braga, Portugal; avicente@deb.uminho.pt; 7 Polymers and Advanced Materials Group (PIMA), School of Technology and Experimental Sciences, Universitat Jaume I (UJI), Avenida de Vicent Sos Baynat s/n, 12071 Castellón, Spain; lcabedo@uji.es

**Keywords:** biopolyesters, nanocellulose, multilayers, compostable, packaging, migration, barrier properties

## Abstract

In the present study, a multilayer, high-barrier, thin blown film based on a polybutylene adipate terephthalate (PBAT) blend with polyhydroxyalkanoate (PHA), and composed of four layers including a cellulose nanocrystal (CNC) barrier layer and an electrospun poly(3-hydroxybutyrate-co-3-hydroxyvalerate) (PHBV) hot-tack layer, was characterized in terms of the surface roughness, surface tension, migration, mechanical and peel performance, barrier properties, and disintegration rate. The results showed that the film exhibited a smooth surface. The overall migration tests showed that the material is suitable to be used as a food contact layer. The addition of the CNC interlayer had a significant effect on the mechanical properties of the system, drastically reducing the elongation at break and, thus, the flexibility of the material. The film containing CNCs and electrospun PHBV hot-tack interlayers exhibited firm but not strong adhesion. However, the multilayer was a good barrier to water vapor (2.4 ± 0.1 × 10^−12^ kg·m^−2^·s^−1^·Pa^−1^), and especially to oxygen (0.5 ± 0.3 × 10^−15^ m^3^·m^−2^·s^−1^·Pa^−1^), the permeance of which was reduced by up to 90% when the CNC layer was added. The multilayer system disintegrated completely in 60 days. All in all, the multilayer system developed resulted in a fully compostable structure with significant potential for use in high-barrier food packaging applications.

## 1. Introduction

Packaging is one of the main strategies for preserving food and maintaining its quality and safety. The objective of food packaging is to satisfy both industrial requirements, i.e., transport and preservation, and customer needs, that is, safety, marketing, and information. The most commonly used materials in food packaging are metals, glass, and paper. However, plastics, since their massive use from the 1950s onwards, have been replacing these other materials thanks to their extraordinary characteristics, such as durability, low cost, and ease of production, as well as their excellent physical and barrier properties [1]. There is a wide variety of polymers in use today, including polyethylene (PE), polyethylene terephthalate (PET), polypropylene (PP), polystyrene (PS), and polyvinyl chloride (PVC), which can be used in a large range of applications including food packaging, both flexible and rigid designs, and trays, bottles, or films [2]. However, these plastics are petroleum-based and non-biodegradable, and their huge use leads to a depletion of natural resources, as well as to an accumulation of waste that causes serious environmental damage [3].

Bioplastics have been claimed to be suitable substitutes for these synthetic plastics. They are of fully or partially renewable origin and can be obtained directly from biomass products, including animals and plants, from conventional synthesis such as poly(lactic acid) (PLA), and from natural/genetically modified organisms including polyhydroxyalkanoates (PHA) [4]. They have attracted attention because of their similarity to conventional plastics as well as their biocompatibility, biodegradability, and non-toxicity, among other characteristics [5]. In this context, PHAs have gained a prominent position due to their biodegradability in different scenarios, i.e., in compost, soil, or aquatic media [6]. Moreover, their physical properties can be modified by changing their monomeric components, which broadens their field of application. Poly(3-hydroxybutyrate) (PHB), for instance, is one of the most studied and widely used PHAs because it has some properties, such as thermal, mechanical, and barrier properties [7], which are very similar to those of conventional plastics such as PP and PE [8]. However, it also has some drawbacks, including brittleness, rigidity, and high crystallinity, which make its industrial processing difficult. Thus, the addition of monomers such as 3-hydroxyvalerate (3HV) or 3-hydroxyhexanoate (HHx) leads to a reduction in crystallinity and an increase in flexibility [9]. Nevertheless, although the use of copolymers can improve some properties, PHAs still have a narrow processing window and are susceptible to thermal degradation during manufacturing processes such as extrusion. For this reason, blending PHAs with other polymers has been used as an alternative to improve some of their mechanical and thermal properties. Thus, when PHAs have been blended with other bio-based biodegradable polymers, such as PLA or chitosan, the materials showed an improvement in ductility, toughness, and thermal stability, as reported by previous authors [10,11]. However, this does not solve the problem of high production costs. Therefore, PHAs have also been blended with biodegradable polymers of petroleum origin, which maintains the biodegradability of the mixture but reduces the costs. Among them, poly(butylene adipate-co-terephthalate) (PBAT) stands out for its degradability in a few weeks by natural enzymes, as well as for its excellent mechanical properties, which improve the processability of the blends [12]. For instance, an increase of up to 500% in the strain at break and about two orders of magnitude in toughness was reported for a PHBV/PBAT blend compared with pure PHBV [13].

In addition to polymer blending, the use of nanoparticles (NPs) has been studied as an alternative or additional method to improve certain characteristics such as transparency, mechanical properties, and barrier properties [14]. Cellulose nanocrystals (CNCs), obtained by acid hydrolysis of cellulosic sources, are one of the most widely used NPs due to their unique properties such as high transparency, reinforcing capacity, and high oxygen barrier [15]. Moreover, they are biodegradable and renewable materials. They have a low aspect ratio, with lengths ranging from 50 to 500 nm and widths of between 5 and 70 nm, and their potential properties are associated with the percolation threshold, where the formation of a specific network with the polymeric matrices occurs [16]. All this makes CNCs perfect candidates for use in combination with biopolymers such as PHAs. Thus, when PHB was blended with CNCs, the nanocomposite showed a 50% and 35% increase in Young’s modulus and tensile strength, respectively [17]. In addition, the nanocomposite exhibited a reduction in water vapor permeability (WVP) because the CNCs resulted in a larger tortuous pathway for water vapor. Similarly, in another study, the addition of CNCs to a PHB matrix resulted in a 52.9% and 46.6% decrease in WVP and oxygen permeability (OP), respectively [18]. However, one of the main drawbacks of these nanocelluloses is that their high gas barrier in the dry state is lost at high relative humidity (RH). Many attempts have been made to make the surface of CNCs hydrophobic using physical and chemical modification methods [19]. However, these modifications sometimes change not only the surface properties but also the original morphology and integrity of the crystal [20]. For this reason, the strategy of using multilayer systems to protect the layer of CNCs between hydrophobic polymers could solve this challenge. In these structures, the outer layers are made of polymers that have proven mechanical and moisture resistance, while the interlayers provide the gas barrier and other properties such as antimicrobial or antioxidant effects [21]. In the field of food packaging, these multilayer systems consist of three to seven layers that meet all requirements and functionalities, but in a lighter and more compact form [22]. Different techniques have been developed to form these multilayer systems such as layer-by-layer, lamination, co-extrusion, and coating techniques, among others [23]. In recent years, electrospinning has emerged as an impressive technique for the development of thin biopolymers layers.

Electrospinning technology focuses on the development of polymeric fibers through the application of electrical and hydrodynamic forces to polymeric solutions. The high voltage causes the appearance of charges on the surface of the solution, which after a certain threshold results in the so-called Taylor cone, which is emitted to the collector in the form of fibers (electrospinning) or particles (electrospraying) [24]. The characteristics of the resulting polymeric material can be modified by adjusting both the parameters of the equipment and the properties of the polymeric solution. Environmental conditions could also affect the final result [25]. In addition to the production of polymeric nanostructures, electrospinning has been used for the encapsulation of active substances due to its high specific surface area and its high encapsulation efficiency, and for the preservation of oxidation- and heat-sensitive ingredients by processing at room temperature [26]. This provides the material with antimicrobial and/or antioxidant properties for a longer period of time [27]. Mechanical and barrier properties can also be improved by the addition of some nanoparticles to the fibers, such as CNCs [28]. Moreover, electrospun fibers can act not only as a film after a thermal post-treatment, but also as a coating layer that can be used as interlayers in multilayer systems [29]. As interlayers, in addition to providing barrier and antimicrobial properties, they can act as adhesive layers, the so-called hot-tack (HT), eliminating the use of synthetic glues [30]. Previous studies developed barrier [31] and barrier and antimicrobial [30] multilayers based on different blown film substrates, in turn based on commercial PHA, containing a CNC layer and an electrospun hot-tack adhesive layer made of PHBV derived from residues. The latter studies focused on, among other PHA-based materials, blends of commercial PHA and PBAT, in which the content of PHA in the blends was of ca. 50 wt.%. This relatively high content of PHA in the blends made the films more difficult to stabilize during the blowing of thin films. Moreover, the latter two studies focused their characterization on the morphology, barrier properties, and antimicrobial properties.

In this context, the objective of this study was to develop a similar compostable multilayer system based on fully compostable materials and characterize additional properties, but in which the PHA content of the substrate was reduced to 30% to increase blowing processability. For this purpose, two outer layers made of a PHB/PBAT blown film (30/70) were used as a mechanical support and water vapor barrier. Moreover, one of these layers was coated with a layer of CNCs to provide the system with a high oxygen barrier. Finally, the system was laminated using a hot-tack made from the electrospun fibers of a PHA derived from fermented cheese whey biowaste. The final multilayer structure was characterized in terms of the surface roughness, surface tension, migration, mechanical properties, adhesion, and barrier properties, as well as the disintegration performance during composting, to assess the necessary requirements for its use in food packaging.

## 2. Materials and Methods

### 2.1. Materials

The PHA blend compound contained 30 wt.% PHB and 70 wt.% PBAT, and 2 parts per hundred resin (PHR) of Joncryl ADR-4400 from BASF (Ludwigshafen am Rhein, Germany) was added to the blend composition as a chain extender to improve processability. The PHB used was Biomer^®^ P309, which was produced by Biomer (Krailling, Germany). This PHB has a melt flow index (MFI) of 10 g/10 min at 180 °C for a load of 2.16 kg. The PBAT, of film-blowing grade and named Ecoflex^®^ F blend C1200, was purchased from BASF (Ludwigshafen am Rhein, Germany). It has an MFI of 2.5–4 g/10 min at 190 °C for a load of 2.16 kg and, according to the manufacturer, it is fully industrial compostable. The manufacture of the blown film blend was carried out as previously reported [30,31], and the processing parameters were optimized as follows: screw speed of 84 rpm, screw pressure of 258 bar, screw temperature profile of 175 °C/175 °C/177 °C/180 °C, superior roll speed of 1.0 m/min, collection roll speed of 2.2 m/min, and tower height of 1500 mm. The final blown film, termed PBAT Blend, had a thickness of about 50 µm and a film width of 250 mm.

A biomass-derived PHBV copolyester was produced using a biowaste of the dairy industry, fermented cheese whey (CW). The 3HV content of the copolymer was 20 mol%. Further information on this material and its production can be found elsewhere [32].

The commercial CNCs were supplied by Melodea Ltd. (Rehovot, Israel), as an aqueous solution with a concentration of 2 wt.% and a pH of 4.5. Chloroform of 99.8% purity was purchased from Panreac S.A. (Barcelona, Spain). 1-Butanol, reagent grade with 99.5% purity, was supplied by Sigma Aldrich S.A. (Madrid, Spain). A food contact primer, Loctite Liofol PR1550, was obtained from Henkel Ibérica S.A. (Barcelona, Spain). This primer is compliant with current food contact regulations, including EFSA, as assured by the manufacturer, and hence its use in food contact applications is permitted. Ethanol absolute (EtOH), ≥99.9% vol., was supplied by Honeywell^®^ (Frankfurt, Germany). Acetic acid (AA) glacial, 99% purity, was supplied by Fisher Chemical^®^ (Loughborough, UK). Methyl methacrylate (MMA) and glycidyl methacrylate (GMA) standards were obtained from Merck Life Science (Darmstadt, Germany).

### 2.2. Application of CNC Coating

Firstly, the PBAT Blend film substrate, pre-processed with a corona treatment (100 watt∙cm^2^/min) to activate the surface in terms of hydrophilicity, was further coated with the food contact primer, which contains a wetting agent to further enhance the spreading and adhesion of water solutions or suspensions on hydrophobic polymer films. The primer was applied in a Rotary Koater (ROKO) Model 30-30-01 device (TMI Machine Testing Inc., New Castle, DE, USA) with a one meter bar head with a 6 µm profile rod, a rolling speed of 2.5 m/min, and a drying temperature of 50 °C. Then, the CNC solution was applied on top of the primer, also on the ROKO machine, using a 50 μm profile rod and a drying temperature of 90 °C during lamination at 2.5 m/min. Two layers of CNC were applied to obtain a complete coating of the PBAT Blend substrate. More details can be found in reference [31].

### 2.3. Electrospun Hot-Tack Layer from Food Waste PHBV

A layer of CW-derived PHBV electrospun fibers was coated directly onto the PBAT Blend substrate as described before [31]. For that purpose, the CW-derived PHBV was dissolved at 8 wt.% in a mixture of chloroform and butanol 75:25 (*w*/*w*) under magnetic stirring for 24 h at 50 °C. The PHBV solution was processed using a high-throughput Fluidnatek^®^ LE-500 pilot line containing a roll-to-roll system (Bioinicia S.L., Valencia, Spain). The device was operated with a motorized multi-needle injector, scanning vertically toward the collectors at 25 °C and 40% RH. The conditions were set at a flow rate of 45 mL/h, 22 kV of voltage, and a 30 cm needle-to-collector distance. An electrospun coating of 5 g/m^2^ grammage was deposited.

### 2.4. Lamination

**Figure 1:**
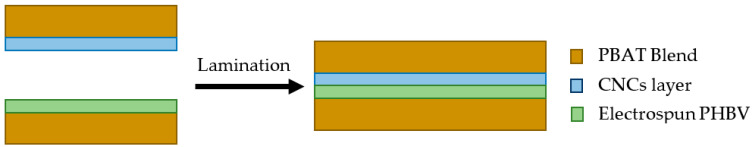
Lamination process of the PBAT Blend with a layer of cellulose nanocrystals (CNCs) and a layer of electrospun poly(3-hydroxybutyrate-*co*-3-hydroxyvalerate) (PHBV) fibers.

The two PBAT Blend substrates, one covered by a CNC layer and the other by a layer of electrospun PHBV fibers, were laminated as previously described [31]. Lamination of both coated layers was performed using a Reliant Powerbond device (Reliant Machinery Ltd., Luton, UK), depositing the samples over the equipment conveyor belt that traveled through the oven at a speed of 5 m/min at 140 °C for 20 s. The thickness of the complete multilayer system, which was opaque, was about 100 µm. Figure 1 displays the final multilayer structure.

### 2.5. Characterization

#### 2.5.1. Confocal Laser Scanning Microscopy (CLSM) and Atomic Force Microscopy (AFM)

The topographic surface of the PBAT Blend monolayer was obtained by 3D confocal laser scanning microscopy (CLSM) (LEXT OLS5000, Olympus, Tokyo, Japan). A MPLAPON50xLEXT objective lens was used, and a 4 × 4 image grid was stitched to achieve a scanning area of 0.9 mm^2^. Multiscan mode was used to obtain at least three scanning areas per sample. Areal roughness was calculated in compliance with ISO 25178-6 [33] by OLS50-BSW Software (Olympus, Tokyo, Japan). Nanoscope III Multimode Atomic Force Microscope (AFM) (Digital Instruments, New York, NY, USA) and DI-TESP probe (42 N/m, 320 kHz, no reflex coating) in tapping mode were used to check confocal results. Profile parameters were measured according to ISO 4287 [34].

Main roughness parameters were determined. The mean roughness (*Sa*) represents the arithmetical mean height deviation of the surface, and it is not affected by extreme points (either high peaks or deep valleys). The root mean square (*RMS*) of the heights of the surface (*Sq*) is the standard deviation of the height distribution and compares the amplitude of peaks and valleys. The maximum height of the surface (*Sz*) is the vertical distance between the deepest valley and highest peak. The Skewness (*Ssk*) is related to the asymmetry of height distribution, and zero (dimensionless) represents normally distributed areas above and below the mean height. The Kurtosis (*Sku*) represents the sharpness of the peaks, and *Sku* = 3 (dimensionless) indicates normally distributed peaks and valleys. Finally, the developed interfacial area ratio of the surface (*Sdr*) is the increment ratio of the topographic area over the definition area.

#### 2.5.2. Surface Tension

The surface tension of the PBAT Blend film with corona discharge treatment (100 watt∙cm^2^/min) was measured and compared with the same film without corona treatment. The equipment was DATAPHYSICS model OCA 35 (Filderstadt, Germany) equipped with an UpUSB 52H camera. The measurement of surface tension was made using 3 solvents of different polarity (water, ethylene glycol, and diiodomethane). Ten measurements were taken per solvent with a variation in the angle less than ±5°.

The surface energy values were also determined by Extended Fowkes method. This method is used to identify surface properties in polymers subjected to corona treatments through the assessment of the interactions between a liquid and solid in terms of dispersive (van der Waals) and polar interactions. In Extended Fowkes method, the hydrogen bond part is also separated out of the polar part.

#### 2.5.3. Migration Test

##### Overall Migration

The overall migration of the multilayer was carried out using the standard procedure of the European Normative EN 1186:2002 [35], which determines the overall migration into food simulants from plastic intended to come into contact with foodstuffs. To demonstrate compliance with the overall migration limit for all type of foods, testing in food simulant A, EtOH 10% (*v*/*v*) in aqueous solution, food simulant B, AA 3% (*w*/*v*) in aqueous solution, and food simulant D2, vegetable oil, was performed. The overall migration test was performed by total immersion (both sides in contact) of the film with a simulation surface/volume ratio of 1 dm^2^/100 mL of simulant at 40 °C for 10 d.

##### Specific Migration

Methyl methacrylate (MMA)-specific migration, potentially arising from the food contact-permitted chain extender used in the blend, was deemed as the most relevant potential migrant within the composition of the multilayer, which is constrained by a specific migration limit. The specific migration limit set by the European Normative EN 13130-1:2004 [36] establishes a maximum migration limit for this chemical, expressed as methacrylic acid (MA) of 6 mg/kg [36]. Three different types of food simulants were used in this test to determine specific migration of MMA: EtOH 10% (*v*/*v*) for foods that have hydrophilic character, AA 3% (*v*/*v*) for foods that have hydrophilic character with pH below 4.5, and EtOH 95% (*v*/*v*) as substitute test media for migration tests of fatty simulant.

Films (0.5 dm^2^) were immersed in 83.33 mL of food simulant over 10 d at 40 °C. For each formulation, specific migration tests were carried out in triplicate, and a blank (jar with food simulant) was also used to check for contaminations. All results were blank subtracted. High-performance liquid chromatography (HPLC) was used to quantify the MMA content in the simulant fluids. A sample of 1 mL of each simulant fluid was transferred to a glass-capped vial. HPLC analysis was performed using an Agilent 1260 Infinity Quaternary LC (Agilent Technologies, Santa Clara, CA, USA) equipped with a Kinetex 2.6 µm C18 100 Å, LC Column (150 × 4.6 mm, Phenomenex, Torrance, CA, USA). The mobile phase was water and acetonitrile (50:50) under a flow rate of 1.0 mL/min, 40 °C, and the injection volume was 10 µL. The compound was eluted and monitored with a Diode Array Detector (DAD) (Agilent Technologies, Santa Clara, CA, USA) at 205 nm, with a retention time of 2.5 min. Then, the area of the peak in the samples was compared with the areas of a calibration curve constructed using a series of MMA standard solutions prepared in each of the simulants at concentrations ranging from 0.001 to 1.000 mg/mL.

#### 2.5.4. Mechanical Tests

The mechanical properties were evaluated by tensile tests using an AG-X 100 Kn Tester from Shimadzu Corporation (Kyoto, Japan) according to UNE-EN ISO 527-3:2018 [37]. Ten measurements were achieved per sample, at room conditions, with a speed of 1 mm/min and a gripping distance of 100 mm. Five sets of specimens were tested parallel to the direction of polymer orientation (MD—machine direction) and five sets perpendicular to the direction of polymer orientation (TD—transverse direction).

#### 2.5.5. Adhesion Properties

A peeling strength test was carried out in the laminated multilayer. The standard method used was the ASTM D1876-08 Standard Test—Method for Peel Resistance of Adhesives (T-Peel Test) [38]. This test method can be used for determining the relative peel resistance of a hot-tack bond between two layers using T-type specimens. These were tested in a universal testing machine (AG-X 100 Kn Tester, Shimadzu, Kyoto, Japan). Five measurements with a width of 15 mm were performed parallel to the direction of polymer orientation (MD), and five other sets, perpendicular to the direction of polymer orientation (TD), were performed at a speed of 100 mm/min and a gripping distance of 150 mm.

#### 2.5.6. Permeance Tests

The water vapor permeance (WVP) of the monolayer and multilayer with and without CNC was determined using the gravimetric method ASTM E398:03 [39] on a tested surface of 5 cm^2^, in duplicate, for 24 h. The equipment was a PERMATRAN-W 398 from Mocon (Minneapolis, MN, USA) used at 30 °C and 90% RH.

The oxygen permeance (OP) coefficient was derived from oxygen transmission rate (OTR) measurements that were recorded using an OXTRAN 2/21 MH (Mocon, Minneapolis, USA). The procedure used was ASTM 3985-2010 [40], where the samples were measured in duplicate with an exposure of 5 cm^2^ at 0% RH and 23 °C.

#### 2.5.7. Industrial Composting Disintegration

The industrial composting disintegration was tested according to ISO 20200:2015 [41]. Test items were cut into 25 × 25 mm pieces and kept in contact with compost substrate. The test was performed in triplicate for each extraction time, and the incubation temperature was continuously kept at 58 ± 2 °C. The total test duration was a maximum of 60 days. The disintegration percentages are based on mass loss of samples over composting time.

### 2.6. Statistical Analysis

Results were evaluated through analysis of variance (ANOVA) using STATGRAPHICS Centurion XVI v 16.1.03 from StatPoint Technologies, Inc. (Warrenton, VA, USA). Fisher’s least significant difference (LSD) was used at the 95% confidence level (*p* < 0.05). Mean values and standard deviations were also reported.

## 3. Results and Discussion

### 3.1. Surface Roughness

**Figure 2:**
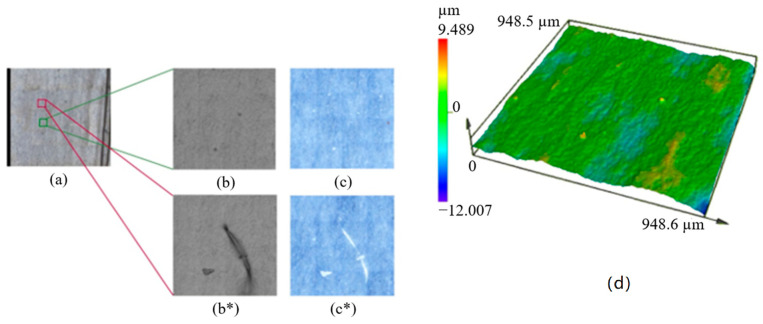
Scanning map (**a**), intensity (**b**), and color (**c**) mode images for the selected area, and 3D surface (**d**) for the PBAT Blend film taken by confocal laser scanning microscopy (CLSM). (b*,c*) highlight some of the few defects spotted in the blown film.

The topographic surface of the substrate film was measured to identify possible irregularities in the outer layer material during processing. The values of the roughness parameters as well as the 3D surface images are presented in Table 1 and Figure 2, respectively. The surface of the film showed some irregular points that were probably formed during the blowing process (Figure 2b*,c*). However, as these defects were isolated and did not represent the real topography of the film, they were not considered in the roughness calculation, but they were considered in the surface description. The negative value obtained for *Ssk*, i.e., −4.9, indicated the predominance of deep valleys rather than peaks, with the presence of some scratches on the surface [42]. For the *Sku* parameter, the sample presented a value of 193.9. This parameter evaluates the randomness of surface heights [43]. Thus, a value higher than three indicated a narrow height distribution and, consequently, a surface with spiked valleys and peaks. Moreover, the lower values of *Sa* and *Sq*, 0.079 and 0.14 µm, respectively, with respect to the higher value of *Sz*, i.e., 12.4 µm, confirmed the presence of some extreme points (peaks and valleys). Finally, the developed surface area over the defined area, represented by the parameter *Sdr*, was not significant, with a value of 4.1%. The additional surface caused by roughness is relevant in packaging technologies because it indicates an increase in the interfacial area where relevant reactions may occur [44].

**Figure 3:**
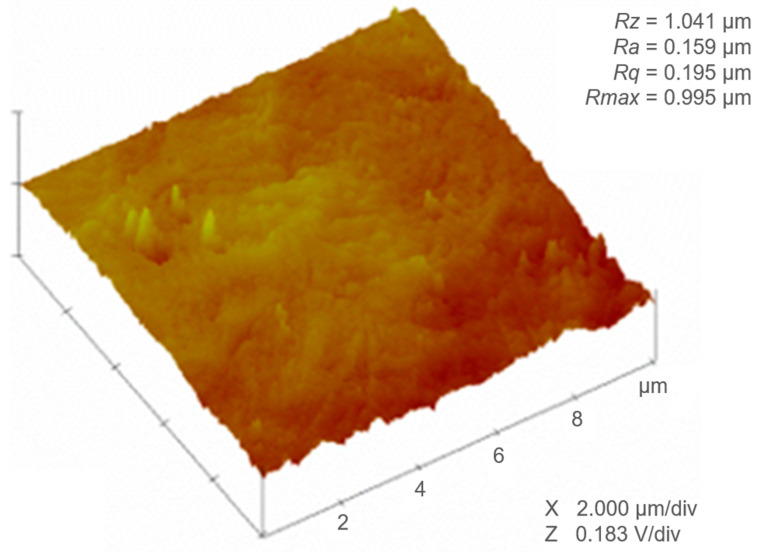
Atomic Force Microscope (AFM) 3D topographic images of 10 µm × 10 µm scanning areas of PBAT Blend film. Profile parameters: arithmetic mean value of the single roughness depths of consecutive sampling lengths (*Rz*), mean profile roughness (*Ra*), root mean square height (*Rq*), and maximum height (*Rmax*).

CLSM was chosen as a non-contact and non-destructive method for topographic measurement with submicron resolution. To verify the results obtained with the CLSM, AFM was used to determine profile roughness parameters and topographic surfaces, as depicted in Figure 3. The parameters *Ra* and *Sa* are correlated with each other. In this case, the *Ra* value was of the same magnitude as the corresponding *Sa* value shown previously in Table 1. It has been reported that as the similarity decreases, the heterogeneity of the sample surface increases [45]. The standard deviation of the heights (*Rq*) and the vertical distance between the deepest valley and the highest peak (*Rz*) were not comparable. These great differences may be mainly due to the fact that the areas scanned by AFM are much smaller than those scanned by CLSM. Thus, in this sense, CLSM analyses seem to be more representative and more reliable for describing the surface. Finally, despite some outliers, the sample may be considered very smooth. The predominance of valleys rather than peaks confers more wear resistance and decreases the probability of scratching and surface loss.

### 3.2. Surface Tension

The surface tension of the inner side of the substrate layer film was tested before and after corona treatment (100 watt∙cm^2^/min). The corona treatment was carried out to activate the layer before deposition of the CNC oxygen barrier layer. Three different solvents were used, namely water, ethylene glycol, and diiodomethane, and the values obtained are presented in Table 2. It can be observed that for both water and ethylene glycol, the contact angle decreased when the corona treatment was applied to the film surface. Thus, for water, the material presented a contact angle of 73.9° without corona treatment, while after surface treatment a contact angle of 53.2° was obtained. Similarly, for ethylene glycol, the material showed contact angles of 53.2° and 24.5° for the untreated and corona-treated film, respectively. On the other hand, a different behavior was observed when testing with diiodomethane, with an increase in the contact angle when the treatment was applied to the surface from 31.6° to 46°. From these trials, it can be concluded that the surface treatment significantly enhanced the wetting properties of the surface with water suspensions such as a CNC water-based suspension, since according to the literature, the smaller the water contact angle, the more hydrophilic the surface becomes [46].

In terms of the surface tension values, determined by the Extended Fowkes method with the contact angle values obtained, the corona treatment resulted in an increase in the surface energy (SE) from 52.8 to 55.5 mN/m. The increase in SE implies an improvement in hydrophilic properties, thus improving the wettability of the surface [47]. This is consistent with the decrease in the water contact angle observed above. Corona discharge treatment introduces oxygen-containing polar groups on the surfaces of the modified films [48]. Therefore, the increase in wettability is also related to the polar groups formed, in this case by the hydrogen bonds, as shown in Table 2, with the values increasing from 3.0 to 15.6 mN/m when the surface was treated. This increase in wettability after corona treatment is in agreement with previous reports in the literature [49].

### 3.3. Migration Assessment of the Film

The overall migration of the multilayer film was tested in three different food simulants to evaluate its performance in all food types. The film was fully immersed in the three simulants studied, i.e., EtOH 10% (*v*/*v*), AA 3% (*w*/*v*), and vegetable oil, at 40 °C for 10 d. The material showed similar behavior with the three simulants, with values of 1.7 mg/dm^2^ for EtOH and AA, and 2.5 mg/dm^2^ for olive oil (as shown in Table 3). In all three cases, the values obtained were below the regulatory limit of 10 mg/dm^2^. Therefore, it can be concluded that, in the overall migration, the multilayer can be used as a food contact layer for all types of foods.

Specific migration of methacrylic acid (MA) monomers on the PBAT monolayer film was performed in different food simulants (Table 4). There is a list of monomers, starting substances, and additives that are regulated by Commission Regulation (EU) No. 10/2011 for use in the manufacture of plastic materials and articles. MA is authorized to be used as an additive in plastic, as established in Annex 1, with a specific migration limit (SML) of 6 mg/kg. MMA is used as an additive for the manufacture of various food contact materials, such as polymethyl methacrylate (PMMA), PET, polyamide (PA), and polycarbonate (PC). Both acrylic ester monomers and MA monomer can migrate from packaging materials into foods [50].

In this study, it was observed that MA migration was higher for hydrophilic simulants, with values of 10.7 and 7.49 mg/kg for 10% EtOH and 3% AA, respectively, which are above the SML, i.e., more than 6 mg/kg. However, for the hydrophobic simulant (95% EtOH), the MA detected was in accordance with the legislation, with a value of 0.63 mg/kg.

### 3.4. Mechanical Properties

The mechanical properties of the PBAT Blend monolayer and multilayer were assessed in terms of the elastic modulus (E), tensile strength (σ_y_), and elongation at break (ε_b_), both in the machine direction (MD) and in the transversal direction (TD), and the results are gathered in Table 5. The monolayer showed a ductile and flexible mechanical performance, with a tensile strength of 20.3 and 17.8 MPa and an elongation at break of 330 and 243% in the MD and TD, respectively. The high values of σ_y_ and ε_b_ in the MD could be attributed to the orientation of molecular chains along that direction [51]. At the same time, the sample showed relatively low values of elastic modulus of around 1.3 GPa when compared with multilayers prepared with pure PHBV and CNC [31]. PBAT is a very flexible material with an exceptionally high ε_b_ of around 700% [52], so its incorporation in a blend results in a toughening of the material. In the present work, the dominant PBAT phase reduced the brittleness and stiffness of the PHA [53], showing higher ε_b_ values than those of other reported PBAT/PHA blends with lower PBAT content [30].

When the multilayer was developed, which contained an interlayer of CNC, the E and σ_y_ values did not significantly change with respect to the monolayer. However, a considerable reduction in elongation at break occurred, with values of 27 and 7.6% in MD and TD, respectively. This high decrease of 91.7% (MD) and 96.9% (TD) is ascribed to the rigidity imposed by the CNC layer to the multilayer structure and is also an indication of the good interlayer adhesion. It has been largely reported in the literature that the use of CNCs in polymeric matrices results in increased fragility [54,55]. Thus, similar results were previously reported in our laboratory when a CNC coating was added to a PHA multilayer blend [30,31].

### 3.5. Peeling Strength

The adhesion capacity of the multilayer with the HT and CNC interlayers was assessed by T-peel adhesion tests both in MD and TD. The results obtained, shown in Table 6, were similar in both directions with a peeling load of 0.082 N and a T-peel strength of 0.006 N/mm. It can be seen that the relative peel resistance of the adhesive bond between the flexible thin films with a layer of CNCs was firmed but not very strong. For example, a previous study in which CNCs were added to a strong commercial adhesive to improve adhesion properties, reported values depending on composition between 0.05 and 0.25 N/mm [56]. This is related to the fact that the smoothness of both the film and the CNC coating was not optimal and made it difficult to achieve a strong interlayer adhesion on the multilayer.

### 3.6. Barrier Properties

The permeance to water vapor (WVP) and oxygen (OP) of the PBAT Blend monolayer, and of the multilayers with and without CNCs, was measured to evaluate their barrier performance. Table 7 shows the thickness and permeance values in terms of WVP and OP. For WVP, the three materials presented with similar values, which ranged from 2.0 to 3.6 × 10^−12^ kg·m^−2^·s^−1^·Pa^−1^, with a slight reduction in permeance in multilayers. [30,31] The addition of a coating of CNC did not significantly affect WVP, despite the hydrophilic nature of this nanostructured layer, because it was protected between the outer water barrier layers. In general, all three materials showed an optimum water vapor barrier similar to commercial materials [57,58]. Thus, while the multilayer developed here exhibited water vapor permeability (permeance x overall thickness) at 30 °C and an 90%RH of ca. 250 × 10^−18^ kg·m·m^−2^·s^−1^·Pa^−1^, a commercial polyvinylidene chloride (PVDC) film presents with water vapor permeability at 38 °C and an 90%RH of 197 × 10^−18^ kg·m·m^−2^·s^−1^·Pa^−1^ [57], and a bio-HDPE (high-density polyethylene)/CNF (cellulose nanofibrils)/bio-LDPE (low-density polyethylene) multilayer presents with a barrier of 556 × 10^−18^ kg·m·m^−2^·s^−1^·Pa^−1^ [58].

In relation to OP, a clear difference can be seen between the three materials. The monolayer film showed an OP of 9.3 × 10^−15^ m^3^·m^−2^·s^−1^·Pa^−1^ while the multilayer without CNCs reduced the permeance to 5.9 × 10^−15^ m^3^·m^−2^·s^−1^·Pa^−1^. However, the greatest difference was achieved when the CNC coating was added, which resulted in a permeance of 0.5 × 10^−15^ m^3^·m^−2^·s^−1^·Pa^−1^. The reduction of more than 90% of OP is due to the high oxygen barrier provided by CNC, which is better than wet ethylene vinyl alcohol (EVOH) and similar to dry polyamide 6 (PA6) [57]. CNC coating of polymeric matrices has been reported to improve the barrier properties of these materials. For example, a PP film coated with CNCs showed an oxygen barrier improvement of 92% [59]. 

### 3.7. Disintegration Tests

**Figure 4:**
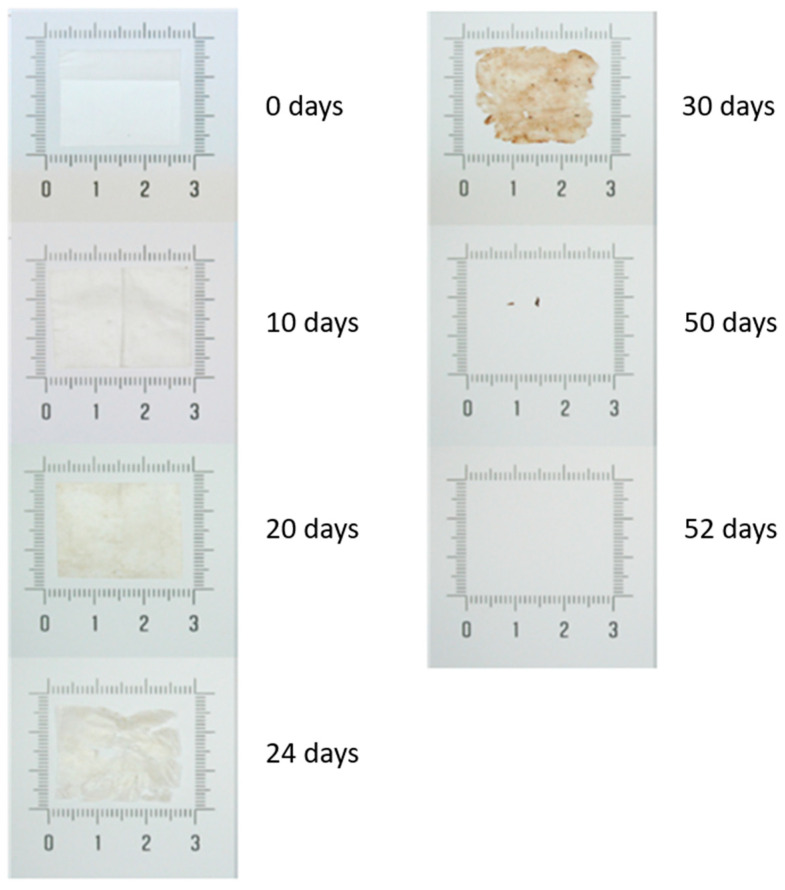
Visual progress of disintegration over time of PBAT Blend film monolayer.

The industrial composting disintegration of the PBAT Blend film was carried out in a laboratory-scale test. For this purpose, the material was kept at 58 °C for 55 d in contact with the compost substrate, as described in the ISO standard, to assess its disintegration rate. Figure 4 shows the visual disintegration process of the PBAT Blend monolayer. It can be observed that after 52 days, there was 100% disintegration of the material, making it suitable for use in food packaging that requires a short disposal time, such as single-use products.

**Figure 5:**
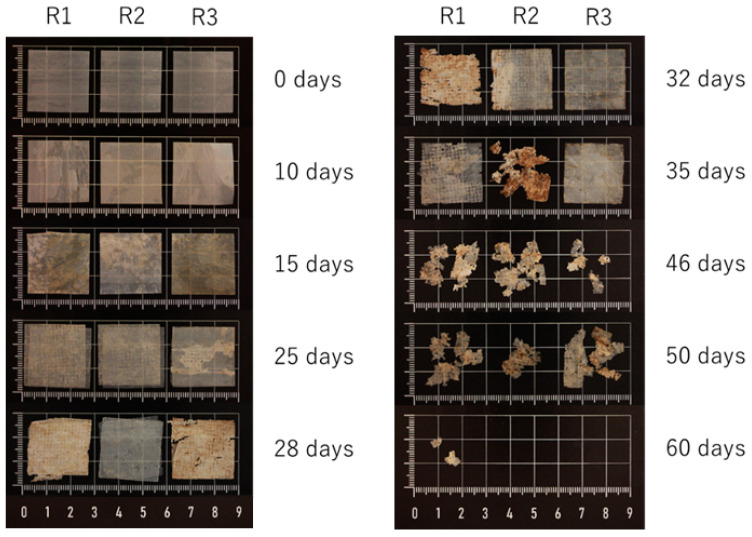
Visual progress of disintegration over time of the multilayer.

The disintegration test was also carried out on the multilayer. The results showed 100% degradation 60 days after the start of the test, with an exponential progression (Table 8). The visual progress of the multilayer is shown in Figure 5. In addition, the dry solids and volatiles obtained were measured. The results showed a total of 24.03% dry solids and 86.22% volatiles.

## 4. Conclusions

In this study, a high-barrier compostable multilayer system was developed to produce a sustainable food packaging film. For this purpose, opaque structural outer layers made of a PBAT/PHBV blend, the so-called PBAT Blend, produced by blown extrusion were used as outer layers. In addition, interlayers of both CNCs and electrospun biomass-derived PHBV acting as a hot-tack were applied. The results showed that the PBAT Blend monolayer exhibited a smooth surface but with some flaws that occurred during the pilot scale film blowing production, but there was not enough wettability for coating of the water-based CNC layer. This hydrophilicity was improved by corona treatment of the surface. The dispersion and adhesion of the CNC water-based suspension was best promoted by adding a food contact primer to the corona-treated film. The addition of the CNC layer produced changes in the mechanical properties of the multilayer compared with those of the PBAT Blend layer, greatly reducing its elongation at break by more than 90% and, therefore, the flexibility and toughness of the material. The adhesive properties provided by the electrospun fibers deposited over one side of the PBAT Blend after lamination to another layer of the PBAT Blend coated with the CNCs were sufficient to generate firm adhesion between the layers, but this was not very strong. However, the addition of the CNC layer significantly increased the barrier properties of the multilayer, resulting in a reduction of 95% in permeance. Finally, overall and some specific migration tests confirmed that the material could be used as a food contact film and disintegration studies showed complete disintegration of both the PBAT-based layer and the multilayer system in less than 60 days.

From the above results, it can be concluded that the film developed in this study has significant potential in food packaging applications as a lidding film to preserve oxygen-sensitive foodstuffs.

## Figures and Tables

**Table 1:** Surface roughness parameters for the substrate PBAT Blend film.

Sample	*L*-Filter (µm)	*Sq* (µm)	*Sa* (µm)	*Sz* (µm)	*Ssk*	*Sku*	*Sdr* (%)
Film	10	0.14 ± 0.01	0.079 ± 0.002	12.4 ± 2.3	−4.9 ± 3.6	193.9 ± 96.5	4.1 ± 0.6

*L*-filter is the Gaussian filter for removal of waviness, *Sq* is the root mean square height of the surface, *Sa* is the arithmetical mean height of the surface, *Sz* is the maximum height of the surface, *Ssk* is the Skewness of height distribution, *Sku* is the Kurtosis of height distribution, and *Sdr* is the developed area ratio.

**Table 2:** Solvent contact angle and surface tension measurements for the PBAT Blend film with and without corona treatment.

		Samples	
		Film without Surface Treatment	Film with Surface Treatment
**Contact angle**	**Θ (^o^) Water**	73.9 ± 0.7 ^a^	53.2 ± 1.0 ^b^
	**Θ (^o^) Ethylene glycol**	53.2 ± 0.8 ^a^	24.5 ± 1.1 ^b^
	**Θ (^o^) Diiodomethane**	31.6 ± 1.6 ^a^	46.0 ± 1.2 ^b^
**Surface tension**	**Surface energy (mN/m)**	52.8 ± 0.2 ^a^	55.5 ± 0.3 ^b^
	**Dispersive (mN/m)**	31.7 ± 0.6 ^a^	27.8 ± 0.5 ^b^
	**Polar (mN/m)**	18.2 ± 0.4 ^a^	12.0 ± 0.3 ^b^
	**H-H (mN/m)**	3.0 ± 0.1 ^a^	15.6 ± 0.3 ^b^

^a,b^ Different letters in the same column indicate a significant difference among the samples (*p* < 0.05).

**Table 3:** Overall mean migration of the multilayer film.

Sample	Test (40 °C, 10 Days)	Result (mg/dm^2^)
Multilayer film	Migration in ethanol 10% (A)	1.7 ± 0.6
	Migration in acetic acid 3% (B)	1.7 ± 0.6
	Migration in olive oil (D2)	2.5 ± 0.7

**Table 4:** Methyl methacrylate (MMA) expressed as methacrylic acid (MA) migrated from the monolayer and multilayer films into different food simulants after the contact condition of 10 days at 40 °C.

Sample	Food Simulants					
	10% (*v*/*v*) Ethanol/Water		3% (*v*/*v*) Acetic Acid/Water		95% (*v*/*v*) Ethanol/Water *	
	(mg/kg)	(mg/g Film)	(mg/kg)	(mg/g Film)	(mg/kg)	(mg/g Film)
PBAT film	10.70 ± 2.17	3.74 ± 1.12	7.49 ± 6.34	3.24 ± 1.53	0.63 ± 1.00	0.49 ± 0.25
Multilayer film	ND	ND	ND	ND	ND	ND

* This was used to replace vegetable oil since it was not possible to make the identification/quantification of the compounds as suggested in the European standard EN13130-1:2004 [36] with this food simulant. ND, not detectable.

**Table 5:** Mechanical properties in terms of elastic modulus (E), tensile strength at yield (σ_y_), elongation at break (ε_b_) of PBAT Blend monolayer and multilayer with cellulose nanocrystals (CNCs) in machine direction (MD) and transverse direction (TD).

Sample	MD				TD		
	Thickness (mm)	E (MPa)	σ_y_ (MPa)	ε_b_ (%)	E (MPa)	σ_y_ (MPa)	ε_b_ (%)
Monolayer	0.05	1270 ± 64 ^a^	20.3 ± 1.3 ^a^	330 ± 22 ^a^	1030 ± 87 ^a^	17.8 ± 0.3 ^a^	243 ± 19 ^a^
Multilayer	0.105	950 ± 64 ^b^	20.6 ± 1.7 ^a^	27 ± 20 ^b^	1030 ± 64 ^a^	14.3 ± 0.5 ^b^	7.6 ± 2.3 ^b^

^a,b^ Different letters in the same column indicate a significant difference among the samples (*p* < 0.05).

**Table 6:** Peeling properties of the multilayer with cellulose nanocrystals (CNCs) in machine direction (MD) and transverse direction (TD).

Machine Direction	Peeling Load (N)	T-Peel Strength (N/mm)
**MD**	0.083 ± 0.024 ^a^	0.006 ± 0.002 ^a^
**TD**	0.081 ± 0.009 ^a^	0.005 ± 0.001 ^a^

^a^ Different letters in the same column indicate a significant difference among the samples (*p* < 0.05).

**Table 7:** Thickness and permeance values in terms of water vapor permeance (WVP) and oxygen permeance (OP) of polyhydroxybutyrate (PHB) Blend film monolayer and multilayer with and without cellulose nanocrystals (CNCs).

		Permeance	
Sample	Thickness (mm)	WVP × 10^12^ (kg·m^−2^·s^−1^·Pa^−1^)	OP × 10^15^ (m^3^·m^−2^·s^−1^·Pa^−1^)
Monolayer PBAT Blend	0.045	3.6 ± 0.3 ^a^	9.3 ± 0.1 ^a^
Multilayer PBAT Blend with hot-tack	0.107	2.0 ± 0.6 ^b^	5.9 ± 0.4 ^b^
Multilayer PBAT Blend with hot-tack and CNCs	0.105	2.4 ± 0.1 ^b^	0.5 ± 0.3 ^c^

^a–c^ Different letters in the same column indicate a significant difference among the samples (*p* < 0.05).

**Table 8:** Percentage disintegration of the multilayer from start to 60 days.

Time (Days)	Disintegration (%)
0	0.0 ± 0.0
10	−1.8 ± 0.2
15	2.8 ± 3.7
21	18.3 ± 7.5
23	24.6 ± 7.5
25	30.5 ± 0.7
28	47.9 ± 7.4
30	49.8 ± 5.7
32	53.4 ± 14.1
35	56.8 ± 10.6
37	58.7 ± 11.2
46	84.5 ± 5.2
50	82.7 ± 6.8
60	99.9 ± 0.2

## Data Availability

Data are contained within the article.
